# Insanity a Symptom of Uterine Disease

**Published:** 1880-05-20

**Authors:** 


### *INSANITY A SYMPTOM OF UTERINE DISEASE.*

Dr. J. G. Meachem, of Racine, Wis., reports in the Chicago Med. Gazette, two cases of insanity caused by ulceration of the cervix uteri, and cured by appropriate treatment directed to that condition.

A few years ago, he says, I received a letter from a lady in the Northern part of this State, asking me if I could do anything to relieve friend of hers who was insane. She gave me as a history that the patient was a female not far from forty years of age. Her insanity came on somewhat suddenly a few weeks after a premature confinement (six months' term), and had lasted nearly a year, some months of which time she had spent in one of our State Hospitals for insane. She had been removed, however, contrary to the wishes of the medical officers of the institution. She had had a good deal of treatment other than at the hospital, all of which resulted in little or no good. My answer to my inquiring friend was that I must see the patient in order to be able to give an opinion at all reliable; that the causes of insanity were many and various; but this case, coming on at the period mentioned, might have some connection with uterine disease, and if so, reasonable hope existed for recovery.

In about two weeks from my writing the patient was brought to my office, with two attendants, She was quite wild at times, and at others quiet and gloomy. It was with some effort that we prevailed upon her to submit to an examination, but after a little it was satisfactorily accomplished. The speculum revealed a most extensive ulceration of the cervix extending far up within the os. This quite convinced me that we had found the key to the difficulty, as it was not the first case of the kind that I had met with. I at once applied a strong solution of nitrate of silver, to which was added two or three drops of nitric acid. The cauterization was most thorough, but the parts were well washed with warm soft water afterward. The day following, she was more like her former self than she had been for months.

I prescribed for her.

℞ Quiniae and ferri citrat........................................ ʒ ij.

Vin. porten............................................................ ℥ vj.

Dose, a teaspoonful before each meal.

℞ Mass. hyd. ........................................................... ℈ j.
Ext. hyosciam........................................................ gr. v.
" nucis vomicae................................................... gr. jss. M.
Ft. pil. No. v.

Dose, one to be taken an hour after dinner.

℞ Potass. bromid...................................................... ℥ j.

Syr. simplicis......................................................... ℥ ij. M.

Dose, a teaspoonful at bed-time.

She remained in the city under my care for two months, and after the first three weeks came regularly to my office by herself, so rapidly had she improved. She returned to her home a little sooner than I desired her to, but she promised to come again to Racine if she found the least necessity for it. She had been separated from her family so long, as she said, for more than a year (the period of her insanity being a blank), and that she was now so well that she could not see it her duty to remain away longer. I will state that, some time before coming to me, she had made several attempts to destroy herself. She had a kind husband and several loving children, but with whom she had had no comfort during the continuance of her insanity, nor they with her, as there was a mutual fear lest one party might do some desperate act of violence to the other. She did not return for further treatment, for she has ever since been well and most happy with her family, and her gratitude has been equal to her happiness.

Another case somewhat similar is that of Mrs. H., a German, of Kenosha county, in this State, aged 38 years. She was the mother of several children, and they had come along in rather quick succession, besides being obliged to do a good deal of outdoor work on the farm (her husband being a farmer), her physical powers had become considerably reduced. She became morose, despondent, and finally, at intervals, perfectly insane. This condition had continued for three months, but during the whole period she had no medical treatment. She became so bad that the husband was obliged to make application for her admission to the insane hospital, and called upon me to visit her for the purpose of certifying to her insanity. After seeing her and getting her history, as stated above, I suggested to her husband that she might possibly be suffering from uterine disease, and before sending her off I would like to make an examination, as perhaps she might be cured at home. After some solicitation and management with the patient, I succeeded, and found almost the exact condition as in case first. Similar treatment, of two months' continuance, resulted in a perfect cure, and to-day she is a hearty, vigorous woman.

The insanity in both of these cases was symptomatic of extensive uterine ulceration.

Is not this condition many times overlooked in the treatment of insane females, even at our public hospitals, on account of the great difficulty of making satisfactory examinations of such patients as often as is necessary for their successful treatment?

